# Validation of the 60-Item Brief COPE for Assessing Coping Mechanisms Among Informal Caregivers of Patients with Cancer in Uganda

**DOI:** 10.5334/aogh.4948

**Published:** 2026-01-30

**Authors:** Rachel Kansiime, Jackson Oryem, Nixon Niyonzima, Milton Mutto, Godfrey Zari Rukundo, Simon Kizito, Joyeeta Talukdar

**Affiliations:** 1 Uganda Cancer Institute, Paediatric Haematology-Oncology Service, Mulago Specialised Hospital, Kampala, Uganda; 2 Uganda Cancer Institute, Kampala, Uganda; 3 Pincer Training and Research Institute, Kampala, Uganda; 4 Mbarara University of Science and Technology, Mbarara, Uganda; 5 Makerere University, School of Psychology, College of Humanities, Kampala, Uganda; 6 Department of Biochemistry, All India Institute of Medical Sciences, New Delhi, India

**Keywords:** coping, caregiving, patients, chronic, structure, instrument, support

## Abstract

The psychological and emotional burdens experienced by informal caregivers of cancer patients highlight the need for effective coping strategies to maintain their well-being. The Brief COPE is a validated questionnaire consisting of 60 items, aimed at evaluating an individual’s coping strategies and is effective in providing a comprehensive profile of responses to stress. This study examined the psychometric properties of the 60-item Brief COPE among informal caregivers of cancer patients in Uganda. A cross-sectional survey involving 200 caregivers was conducted, during which participants completed the Brief COPE alongside measures of psychological distress. Internal consistency was evaluated using Cronbach’s α, while test–retest reliability was assessed to determine temporal stability. Construct validity was examined through confirmatory factor analysis (CFA) and correlational analysis with related constructs. The Brief COPE demonstrated excellent internal consistency (Cronbach’s α = 0.92) and strong test–retest reliability (*r* = 0.89). The anticipated factor structure was validated through CFA, and significant correlational analysis corroborated its construct validity. These results affirm that the 60-item Brief COPE is a reliable and valid tool for assessing the coping strategies of informal caregivers of cancer patients in Uganda.

## Introduction

Informal caregivers for cancer patients often experience significant emotional strain and stress. Their coping strategies are crucial for managing the practical and emotional challenges associated with caregiving. The Brief COPE, a widely used tool for assessing coping strategies, has been validated in several populations; however, it has not been extensively studied among informal caregivers in Uganda. This research aims to assess the validity and reliability of the 60-item Brief COPE as a measure of coping mechanisms in this context. The Brief COPE inventory, developed by Carver in 1997, is a well-known self-report tool that evaluates a broad spectrum of coping responses. Its 60-item version offers a comprehensive assessment of coping mechanisms across various demographics and medical conditions. Although it is widely utilized internationally, the cultural relevance and psychometric properties of the Brief COPE have been investigated in different contexts, including Uganda, where caregiving environments and stressors may vary considerably from those in Western populations.

Recent studies conducted in Uganda have employed the Brief COPE to examine how various groups of patients and their caregivers manage stress. For instance, Kaggwa et al. [1] investigated the coping mechanisms of caregivers for individuals with HIV/AIDS, demonstrating that the tool is applicable in Uganda. Similarly, Ndyanabangi et al. [2] assessed the Brief COPE among caregivers of patients with chronic illnesses in Uganda, concluding that it is both reliable and valid. Given the cultural distinctions and unique caregiving roles present in Uganda, it is crucial to validate the 60-item Brief COPE specifically for informal caregivers of cancer patients to ensure its reliability and validity. Such validation will facilitate the development of targeted psychosocial interventions aimed at enhancing caregiver resilience, ultimately leading to improved treatment outcomes for cancer patients. This research seeks to validate the 60-item Brief COPE within this specific context, focusing on its reliability and validity as a tool for measuring coping strategies.

## Literature Review

1. **Coping mechanisms in caregiving**
  Informal caregivers, who are typically family members or friends providing unpaid assistance, frequently encounter significant stress. Research indicates that effective coping mechanisms are essential for alleviating this stress and preserving the mental health of caregivers [3]. Common strategies for coping encompass problem-solving, seeking social support, and emotional regulation [4].
2. **The Brief COPE instrument**
  The Brief COPE is a 60-item instrument created to evaluate a variety of coping strategies. It comprises 15 subscales, each corresponding to a distinct coping mechanism [5]. This tool has been extensively validated across diverse populations, including individuals with chronic illnesses and caregivers [6, 7]. While the reliability and validity of the Brief COPE have been confirmed in various cultural contexts, its use in Uganda necessitates specific validation.
3. **Coping mechanisms among African caregivers**
  Research on coping strategies among African caregivers, particularly in Uganda, reveals that cultural elements significantly influence how these caregivers handle stress [8]. Factors such as traditional beliefs, community support networks, and socioeconomic conditions shape their coping methods [9]. It is vital to comprehend these cultural intricacies for a precise evaluation of coping mechanisms.
4. **Validation of psychological instruments in Uganda**
  The process of validating psychological tools in Uganda has been somewhat limited; however, it is essential for confirming that these instruments are both culturally appropriate and psychometrically valid [10]. Tools such as the Brief COPE need to be modified and validated within local contexts to guarantee their efficacy in measuring psychological constructs.

## Methods

### Participants

In Uganda, it is common for family members or acquaintances to offer unpaid care to cancer patients through informal caregiving, which imposes a considerable emotional and psychological strain on the caregiver [3]. Research indicates that coping strategies such as problem-solving, seeking social support, and managing emotions are crucial for effectively dealing with this stress [4]. To address this concern, our study recruited 200 unpaid caregivers from two major cancer treatment centers in Uganda. To be eligible, participants needed to have provided care for a cancer patient for no less than six months and to be at least 18 years old. Prior to enrollment, all participants submitted written informed consent in line with the ethical standards of research. Additionally, the researchers secured ethical approval from the research and ethics committee of MUST and administrative clearance from the research and ethics committee of the Uganda Cancer Institute.

### Measures

The Brief COPE, a widely used self-report instrument consisting of 60 items, is designed to assess a diverse range of coping strategies [5]. This tool encompasses 15 subscales that evaluate various coping dimensions, including active coping, denial, emotional support, planning, humor, acceptance, religion, substance use, venting, self-blame, among others [6, 11]. While research has demonstrated its validity and reliability across different cultural contexts, specific validation studies in Uganda remain limited [10]. Considering the unique cultural factors that influence coping mechanisms in African environments, such as traditions and social support systems [8, 9], this study sought to confirm the continued reliability of the Brief COPE as a psychometric tool for informal caregivers in Uganda.

### Procedure

In order to enhance cultural relevance and understanding, a certified language expert translated the Brief COPE questionnaire into relevant local languages before the data collection process. The instrument was pilot-tested with a small cohort of caregivers (*n* = 10) and underwent a back-translation procedure to ensure the accuracy of the translation. The feedback received was utilized to refine the wording as needed. After being recruited from two major cancer treatment centers, specifically the Uganda Cancer Institute in Kampala City and the Mbarara Regional Cancer Centre located in Mbarara City (Western Uganda), participants were classified based on the facility from which they were recruited. To maintain confidentiality and to create a link between baseline and follow-up responses for test–retest reliability, each participant was assigned a unique identification code.

To reduce distractions and ensure privacy, the self-administered questionnaire was distributed individually in designated private rooms within the treatment facilities. Trained research assistants provided standardized instructions and were available during the session to address questions, clarify any ambiguities, and assist participants with limited literacy, all while ensuring that their responses remained unbiased. Participants were encouraged to complete the questionnaire independently. A subgroup of 30 caregivers was asked to fill out the same questionnaire a second time, two weeks after the initial administration, to assess test–retest reliability. Once completed, all forms were collected, checked for completeness, and securely stored for subsequent data entry and analysis. The steps followed in the study are clearly explained in Table 1 below.

**Table 1:** Step-by-step procedure for validating the 60-item brief COPE.

STEP	DESCRIPTION
1. Translation	The Brief COPE was translated into relevant local languages by a certified language expert to ensure cultural relevance and linguistic clarity.
2. Back-translation	The translated version was back-translated into English by an independent expert to verify accuracy and conceptual equivalence.
3. Pilot testing	A pilot test was conducted with a small group of 10 informal caregivers to identify unclear wording and ensure cultural appropriateness. Adjustments were made as needed.
4. Recruitment	200 informal caregivers were recruited from two cancer treatment centers and grouped according to the site. Eligibility criteria were confirmed before enrollment.
5. Consent and ID coding	Written informed consent was obtained. Each participant received a unique identification code to protect confidentiality and track responses for test–retest reliability.
6. Administration	Participants completed the self-administered questionnaire in private rooms within the centers. Trained research assistants provided standardized instructions.
7. Assistance	Research assistants remained available to clarify items or assist participants with reading difficulties, ensuring they did not influence answers.
8. Test–retest subsample	A subgroup of 30 participants was randomly selected to complete the same questionnaire again after two weeks to assess test–retest reliability.
9. Data collection	Completed forms were collected immediately, checked for completeness, and securely stored for data entry and statistical analysis.

## Data Analysis

All gathered data were examined to evaluate the reliability and validity of the 60-item Brief COPE. Internal consistency was assessed using Cronbach’s α for both the overall scale and each of its 15 subscales to ensure reliable measurement of coping dimensions. To investigate the stability of the instrument over time, test–retest reliability was evaluated using Pearson’s correlation coefficient on a subsample of participants who completed the tool on two occasions. Construct validity was assessed through confirmatory factor analysis (CFA) to determine whether the proposed factor structure aligned with the observed data. Furthermore, convergent validity was established by correlating Brief COPE scores with scores from the General Health Questionnaire-12 (GHQ-12) and the Coping Strategies Inventory, thereby providing evidence that the instrument effectively measures coping mechanisms associated with psychological distress among informal caregivers.

## Results

### Sociodemographic characteristics

The sociodemographic data presented in Table 2 offered essential background for the analysis of the psychometric outcomes of the 60-item Brief COPE among Ugandan informal caregivers of cancer patients. Table 2 illustrated that, with a test statistic of 3.181 and a *p*-value of 0.002, there was a statistically significant difference in the mean age between male caregivers (37.3 years, SD = 12.2) and female caregivers (33.5 years, SD = 11.4). This suggests that male family members often assumed caregiving responsibilities, possibly due to cultural or domestic norms where older men acted as guardians or decision-makers in the absence of younger women or when they were already burdened with other tasks. Conversely, the lower average age of female caregivers aligns with global data indicating that women generally undertake caregiving roles earlier in life, often while managing children and other household responsibilities. Male caregivers were found to care for older patients (mean age = 38.0 years, SD = 24.1) in comparison to female caregivers (mean age = 32.0 years, SD = 22.7), with a significant difference in the mean age of the patients based on the caregiver’s gender (*p* = 0.013). This pattern may reflect family dynamics, where female caregivers are more inclined to care for younger dependents, such as children or younger adult family members with cancer, while male caregivers are more likely to provide care for elderly parents or relatives.

**Table 2:** Sociodemographic characteristics of respondents.

VARIABLE	MALE	FEMALE	OVERALL	TEST STAT	*p*-VALUE
Age of caregiver (mean, SD)	37.3 (12.2)	33.5 (11.4)	34.7 (11.8)	3.181	0.002
Age of patient (mean, SD)	38.0 (24.1)	32.0 (22.7)	35.4 (23.6)	2.506	0.013
Occupation (%, *N*)			–	19.114	0.014
Farmer	59 (34.3)	113 (65.7)	173 (39.9)		
Business	32 (28.6)	80 (71.4)	113 (26.0)		
Others	49 (0.0)	98 (100)	1 (0.23)		
Distance to nearest HC (%, *N*)			–	7.298	0.199
Less than 20 km	12164 (37.2)	228,108 (62.7)	349,172 (40.1)		
More than 20 km	18 (24)	57 (76)	75 (17.6)		
Non-response	1 (14.2)	6 (85.7)	7 (1.62)		
Education level (%, *N*)			–	20.730	0.004
Primary level	47 (29.94)	118 (70.0)	157 (36.94)		
Secondary school level	50 (29.31)	97 (70.69)	147 (27.29)		
Completion of certificate course	22 (37.92)	36 (62.07)	58 (13.65)		
University education	11 (40.74)	20 (59.26)	31(6.35)		
Non-response	10	30 (16.7)	40 (1.39)		
Disability (%, *N*)			–	1.702	0.427
No	136 (32.5)	282 (67.5)	420 (97.7)		
Yes	4.0 (40)	6.0 (60)	10 (2.3)		
Non-response	0 (.0)	3.0 (100)	3 (0.69)		
Level income (000) (median, range)	309710.7 (402374.1)	276182.6 (555397.6)	150,000 (300–5,000,000	.588	0.557
Religion (%, *N*)			–	16.417	0.006
Anglican	62 (41.6)	87 (58.4)	152 (36.1)		
Roman Catholic	49 (33.3)	98 (66.7)	147 (34.9)		
Moslem	5 (11.4)	39 (88.6)	44 (10.5)		
Born again	19 (26)	54 (74)	73 (17.3)		
Seventh day Adventist	1 (20)	4 (80)	5 (1.2)		
Non-response	4 (30.7)	9 (69.3)	13 (3.01)		
Type of CA (%, *N*)					
Breast cancer	11 (31.4)	24 (68.6)	35 (8.1)		
Colorectal cancer	6 (40)	9 (60)	16 (3.7)		
Prostate cancer	13 (48.1)	14 (51.8)	27 (6.2)		
Cervical cancer	18 (27.7)	47 (72.3)	65 (15.0)		
Leukemia	28 (35.4)	51 (64.5)	80 (18.5)		
Head and neck Cancer	14 (31.8)	30 (68.2)	45 (10.4)		
Childhood cancer	2 (12.5)	14 (87.5)	16 (3.7)		
Not known	48 (31.6)	102 (68.4)	150 (34.4)		
Stage of CA (%, *N*)			–	10.495	0.033
Early stage	37 (45.1)	45 (54.9)	82 (19.3)		
Late stage	35 (34.3)	67 (65.7)	104 (24.4)		
Terminal stage	11 (31.4)	24 (68.6)	36 (8.5)		
Not known	57 (26.1)	155 (73.9)	213 (47.9)		

One significant factor was occupation (χ² = 19.114, *p* = 0.014). Among both male and female caregivers, farming emerged as the predominant occupation; 59 males and 113 females, accounting for nearly 40% of the sample, identified themselves as farmers. This significant percentage highlighted the rural environment in which many Ugandan caregivers resided, where subsistence farming remained the primary source of income. The considerable gender gap indicated that demanding agricultural work often coexisted with caregiving responsibilities, likely exacerbating the stress on caregivers’ physical and emotional health. Furthermore, women were more inclined than men to engage in business activities (80 compared to 32). The dual roles of managing small enterprises and providing unpaid care may have heightened stress levels and contributed to the formation of coping strategies that predominantly depended on multitasking and informal support networks.

Additionally, there were notable occupational disparities (*p* = 0.014), indicating that caregiving responsibilities were affected by the type of employment. With a considerable gender distribution of 59 men and 113 women, the predominant occupation was farming. It is noteworthy that women were more represented in business roles, highlighting the dual burden many women face in balancing caregiving and earning an income. Occupation plays a crucial role as it can impact available coping resources. For example, individuals involved in informal farming may lack the same access to formal support systems as those who own businesses in urban areas.

Significant gender disparities were observed in educational levels (χ² = 20.730, *p* = 0.004). In total, 36.9% of caregivers achieved primary education, while 27.3% completed secondary education, suggesting that a considerable portion had only reached primary or secondary schooling. Only 6.3% of individuals attained a university degree, indicating that higher education is relatively rare. The absence of formal education may have limited caregivers’ awareness of structured coping therapies or accessible psychosocial support, potentially leading to a greater dependence on religious and community-oriented coping strategies. Males exhibited a slightly higher likelihood than females of holding certificates or university degrees, which could have affected their access to information regarding patient care or their engagement with the healthcare system.

Both male and female caregivers reported modest median earnings of approximately 300,000 UGX, with a significant range spanning from 300 to 5,000,000 UGX. The test statistic (0.588) and *p*-value (0.557) indicated that there were no significant gender differences in income levels. Nevertheless, the overall low median income highlighted the financial vulnerability of caregivers. Financial constraints likely exacerbated psychological distress and hindered access to formal coping resources such as paid support, counseling, or respite care.

Significant gender differences were observed in religious affiliation (χ² = 16.417, *p* = 0.006). The predominant categories of caregivers included Anglican and Roman Catholic, with a greater percentage of male caregivers identified among Anglicans and a higher percentage of female caregivers among Roman Catholics. It is likely that religion played a crucial role in shaping coping strategies, especially in contexts where resilience was primarily sourced from spiritual beliefs and faith-based community support. A dimension evaluated by the Brief COPE’s “religion” subscale indicated that the larger representation of females among “Born Again” Christians and Muslims suggested potential differences in the utilization of religious coping strategies between male and female caregivers.

Despite the absence of a notable gender disparity in the distance to the nearest health center (χ² = 7.298, *p* = 0.199), it remained a significant contextual element. More than 40% of caregivers indicated that they needed to travel over 20 km to access cancer care. This situation imposed further financial and logistical burdens, likely increasing stress levels and impacting their coping strategies, especially regarding planning and seeking necessary support.

Only 2.3% of caregivers overall reported having a disability, with no significant difference observed between males and females (χ² = 1.702, *p* = 0.427). Caregivers with disabilities likely encountered additional challenges that complicated their coping requirements and heightened the demand for supportive interventions, although the limited sample size hindered a comprehensive analysis.

There was no notable difference in the type of cancer based on the gender of the caregiver (χ² = 10.882, *p* = 0.144). According to the national cancer incidence statistics of Uganda, the most common cancers were leukemia (18.5%), cervical cancer (15%), and breast cancer (8.1%). Nevertheless, a significant gender disparity was observed in the cancer stage (χ² = 10.495, *p* = 0.033). The higher number of female caregivers for patients with unknown cancer stages raised concerns that women might have been either deliberately excluded from clinical discussions or had limited access to medical information, potentially heightening anxiety, stress, and the demand for emotional support. The uncertainty stemming from a lack of knowledge regarding a patient’s disease stage often exacerbated the psychological burden on caregivers and affected their coping strategies, such as acceptance, seeking social support, or engaging in religious coping. However, the data presented in Table 2 indicated that the cancer stage of the patient being cared for was significantly linked to the caregiver’s gender, with a test statistic of 10.495 and a *p*-value of 0.033, signifying a statistically significant difference. This finding implied that the stage of the patient’s illness affected caregiving roles differently for males and females within this sample. Among caregivers who acknowledged knowing the stage of the patient’s cancer, the largest proportion was responsible for patients in the late stage (35 males, 67 females). A smaller number of caregivers attended to patients in the early stage (37 males, 45 females) and the terminal stage (11 males, 24 females).

Nevertheless, a remarkable finding was that a significant number of caregivers—57 males and 155 females—indicated they were unaware of the stage of the patient’s cancer. This “not known” category represented nearly half of the total respondents (47.9%), with a notably higher representation of females in this group. The significant gender disparity in the “not known” category underscored potential communication gaps between healthcare providers and family caregivers. Female caregivers were more likely than their male counterparts to be uninformed about the patient’s precise disease stage, which may reflect cultural dynamics where men are often more engaged in direct discussions with medical professionals or serve as family decision-makers. This lack of information could have heightened psychological uncertainty and distress for female caregivers, who typically bear the daily caregiving responsibilities yet may not be directly involved in treatment conversations. From a coping standpoint, uncertainty regarding the cancer stage carries significant implications. When caregivers are deprived of clear information about disease progression, they may face increased anxiety and a reduced sense of control—two elements closely associated with coping mechanisms. In this study, caregivers who were unaware of the cancer stage might have learned more towards emotion-focused coping strategies, such as seeking emotional support, engaging in religious coping, or practicing acceptance and positive reframing to alleviate their stress.

Conversely, caregivers who were aware of the patient’s cancer stage might have been more effectively equipped to implement problem-focused coping strategies, including planning, active coping, and seeking instrumental support. Understanding whether the patient’s condition was at an early, late, or terminal stage could allow caregivers to better anticipate care requirements and mobilize necessary resources, which is consistent with theoretical frameworks of stress and coping. Ultimately, the notable disparity in cancer stage awareness between male and female caregivers highlighted the necessity for clear and inclusive communication among healthcare providers and all family members engaged in caregiving. Providing caregivers with transparent information regarding prognosis and treatment strategies may alleviate uncertainty, enhance coping mechanisms, and support the caregiver’s psychological well-being—results that the validated Brief COPE can effectively assess and track.

Concerning the interaction between caregivers and patients, significant gender differences were observed (χ² = 19.796, *p* = 0.019). The predominant caregivers were female children, followed by spouses and siblings. This finding underscores the cultural expectation that daughters and female relatives should assume the majority of caregiving duties, especially within Uganda’s extended family frameworks. Male spouses were more frequently the primary caregivers compared to their female counterparts, while female siblings outnumbered male siblings in caregiving roles. These patterns may reflect the existing family dynamics and gender roles associated with caregiving arrangements.

There was no statistically significant difference in the duration of care provided by males versus females (χ² = 3.183, *p* = 0.364). This indicates that individuals, regardless of gender, tended to provide care for similar durations after assuming the caregiving role. The importance of long-term coping strategies and support systems for all caregivers, regardless of gender, was further emphasized by this prolonged engagement.

The sociodemographic findings collectively illustrated the complex interactions between gender, age, occupation, income, education, religion, patient relationships, and caregiving dynamics in Uganda. These elements likely affected the selection and implementation of coping strategies by caregivers, as evaluated by the Brief COPE. For example, limited access to formal mental health services may have resulted from low income and educational levels, underscoring the significance of faith-based coping strategies and family support systems. The relevance of the subscales of the Brief COPE in capturing various coping aspects relevant to the caregiving context in Uganda was emphasized by the statistically significant gender differences observed in key variables.

**Figure 1:**
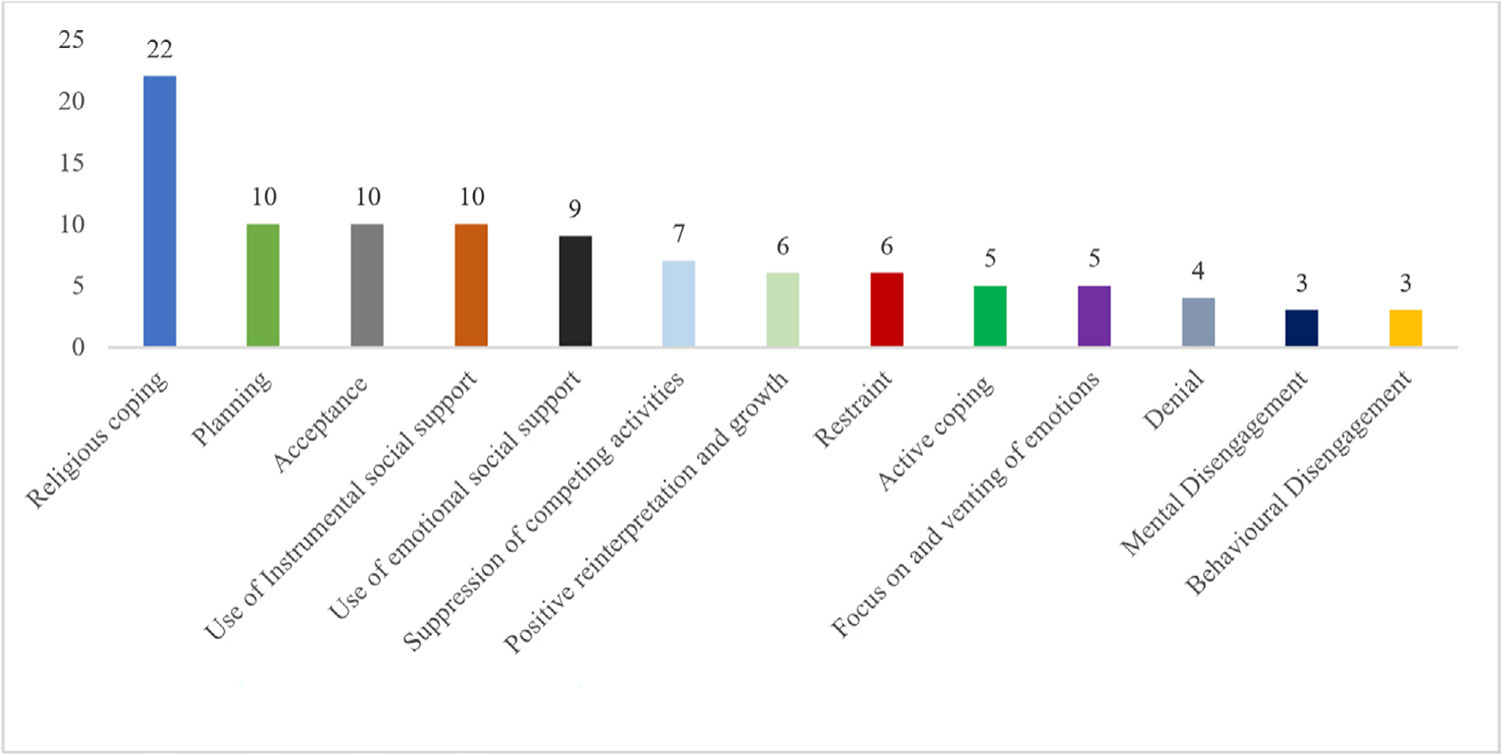
Coping mechanism applied by caregivers.

Figure 1 indicates that the majority of caregivers employed religious coping strategies (22%), followed by those who utilized planning, acceptance, and instrumental social support (10%). In contrast, the smallest proportion of caregivers resorted to mental and behavioral dis-engagement (3%).

### Reliability of the brief COPE

Reliability is an essential psychometric characteristic that reflects the degree to which an instrument consistently measures a construct across various items and over time [12]. In the case of the 60-item Brief COPE, reliability was assessed through two complementary methods: internal consistency and test–retest reliability (Table 3).

**Table 3:** Internal consistency testing results of the COPE instrument.

TOOLS USED	SUBSCALES OF THE TOOLS	NAMES OF THE SUBSCALES	CRONBACH’S α
COPE	Subscale 1 Subscale 2 Subscale 3	Problem-solving Denial Active coping	0.91 0.92 0.91

Internal consistency evaluates whether the items within each subscale consistently measure the same underlying coping strategy. This was measured using Cronbach’s α coefficient, which ranges from 0 to 1. A higher α-value signifies greater internal coherence among items within the subscale. In this study, the internal consistency of the primary subscales was outstanding, with Cronbach’s α-values of 0.91 for problem-solving, 0.92 for denial, and 0.91 for active coping. These values significantly surpassed the conventional threshold of 0.70, which is generally regarded as the minimum acceptable standard for psychological assessments [13]. Alpha coefficients exceeding 0.90 are usually considered excellent, indicating that the items within each subscale are highly interrelated and that each subscale reliably reflects a coherent aspect of coping behavior.

The strong internal consistency found in this sample of Ugandan caregivers indicated that the processes of translation and cultural adaptation maintained the conceptual clarity and semantic integrity of each item. Furthermore, it suggested that informal caregivers interpreted the items in a consistent manner, regardless of possible variations in literacy levels or familiarity with psychological terminology.

Test–retest reliability was employed to evaluate the stability of the Brief COPE over time—specifically, whether the instrument would produce consistent results when administered to the same individuals under similar conditions at different times. To assess this, a subset of caregivers completed the Brief COPE on two occasions, with a two-week gap between the administrations. The resulting Pearson’s correlation coefficient for the total score was 0.89, indicating a strong test–retest reliability. This high correlation implied that the coping responses measured by the Brief COPE represented stable patterns of behavior and attitudes, rather than fleeting mood states or situational influences.

The significant test–retest outcome was particularly crucial considering the dynamic and demanding environment of caregiving for cancer patients, where coping mechanisms may vary in response to alterations in the patient’s health or the caregiver’s personal situation. In spite of these potential daily stressors, the Brief COPE demonstrated strong temporal stability, affirming its appropriateness for application in both cross-sectional and longitudinal research frameworks within this setting.

Additionally, the high reliability estimates for the Brief COPE in this study were consistent with or surpassed the values documented in previous research conducted across various populations. For instance, Carver (1) initially reported Cronbach’s α-values ranging from 0.50 to 0.90 for different subscales of the Brief COPE. Later studies involving caregivers and patients in both Western and non-Western settings have shown similar reliability ranges; however, alpha coefficients exceeding 0.90 are relatively uncommon and indicate very strong internal consistency. Collectively, these results suggest that the 60-item Brief COPE is not only psychometrically robust but also culturally suitable for evaluating coping mechanisms among informal caregivers in Uganda. The high internal consistency and outstanding test–retest reliability endorse its application in clinical practice and research, including studies aimed at tracking coping over time, assessing the effects of psychosocial interventions, or investigating the connection between coping strategies and caregiver well-being. Future research may build upon this evidence by reporting Cronbach’s α-values for all 15 subscales, analyzing inter-item correlations within each subscale, and examining whether specific coping domains exhibit greater or lesser temporal stability within this cultural framework.

### Validity of the brief COPE

Establishing validity is crucial to ensure that a psychological instrument effectively measures the constructs it is designed to evaluate. In the case of the 60-item Brief COPE, construct validity was assessed through CFA, along with the interpretation of factor loadings and uniqueness values, as illustrated in Table 4.

**Table 4:** Factor analysis results of the COPE instrument.

VARIABLES	FACTOR 1	FACTOR 2	FACTOR 3	UNIQUENESS
cope1new	–0.4554	–0.3300	–0.0747	0.6782
cope2new	–0.0749	–0.3468	0.1719	0.8446
cope3new	–0.2491	–0.6694	0.0335	0.4887
cope4new	0.4527	0.2179	0.0896	0.7395
cope5new	0.4488	0.2990	–0.0082	0.7091
cope6new	–0.1968	–0.5000	0.1966	0.6726
cope7new	0.5742	–0.0522	–0.0226	0.6671
cope8new	–0.1180	–0.5648	0.0287	0.6663
cope9new	0.3302	0.4935	0.0440	0.6455
cope10new	0.5373	0.2186	0.2095	0.6196
cope11new	0.5643	0.3253	0.1095	0.5638
cope12new	0.6392	0.1766	0.0809	0.5537
cope13new	–0.5022	–0.2357	0.0270	0.6915
cope14new	0.1737	0.6241	–0.1690	0.5518
cope15new	–0.2524	–0.5826	0.0655	0.5926
cope16new	0.5619	0.0061	–0.0695	0.6794
cope17new	0.5603	0.2653	0.1376	0.5967
cope18new	0.3936	0.3856	0.1355	0.6781
cope19new	–0.2499	–0.5222	–0.1710	0.6356
cope20new	0.5432	0.2294	0.1766	0.6212
cope21new	–0.0523	–0.6014	–0.1325	0.6180
cope22new	0.2511	0.4329	0.1532	0.7260
cope23new	–0.0129	0.6048	–0.1624	0.6076
cope24new	0.3361	0.4173	–0.0998	0.7030
cope25new	0.4205	0.3797	0.0557	0.6759
cope26new	0.4627	0.1300	0.0151	0.7688
cope27new	–0.0520	–0.5594	–0.1294	0.6677
cope28new	0.5038	0.2835	0.1258	0.6500
cope29new	0.4519	0.3183	0.0551	0.6914
cope30new	0.4877	0.2465	0.1394	0.6820
cope31new	–0.1215	–0.6139	–0.0037	0.6084
cope32new	0.2983	0.3055	–0.1571	0.7930
cope33new	0.5930	0.0902	–0.1790	0.6081
cope34new	–0.1201	–0.5213	0.4089	0.5466
cope35new	0.4592	0.2887	–0.0773	0.6998
cope36new	0.5022	0.2417	–0.2856	0.6078
cope37new	0.0685	0.2909	–0.3238	0.8058
cope38new	0.5185	0.1527	–0.0468	0.7056
cope39new	0.5833	0.1601	–0.0059	0.6341
cope40new	–0.3463	–0.3208	0.1487	0.7551
cope41new	0.5170	0.3031	–0.0742	0.6353
cope42new	0.3948	–0.0202	–0.0508	0.8412
cope43new	–0.3585	–0.4176	0.2392	0.6399
cope44new	–0.1453	–0.5623	0.0518	0.6600
cope45new	0.6073	0.3071	–0.0624	0.5330
cope46new	0.5168	0.1727	–0.2020	0.6624
cope47new	–0.4410	–0.2895	0.2272	0.6701
cope48new	0.5245	0.2627	–0.1285	0.6393
cope49new	–0.1640	–0.3377	0.2449	0.7991
cope50new	0.6206	0.0757	–0.1606	0.5833
cope51new	0.6768	0.0146	–0.0989	0.5320
cope52new	0.5995	0.0393	–0.1887	0.6035

Construct validity via CFA: CFA assesses whether the theoretical framework of a tool—specifically, the various coping domains initially identified by Carver [5]—aligns with the observed data from the target population. The CFA conducted in this research investigated whether the items in the Brief COPE were grouped as anticipated to represent coherent coping factors. Table 4 displayed the factor loadings for each COPE item across three extracted factors. These factors likely corresponded to broader coping domains, such as problem-focused coping, emotion-focused coping, and avoidant coping, which are consistent with widely recognized stress and coping frameworks. Factor loadings indicate the strength of the relationship between an item and its underlying factor. Generally, loadings exceeding 0.40 are deemed acceptable, with higher values signifying stronger contributions to the factor. In this research, numerous items exhibited strong loadings on their respective factors. For example:

- cope12new had a factor loading of 0.6392 on factor 1, suggesting a solid association with that coping domain.
- cope51new similarly loaded 0.6768 on factor 1, reinforcing the internal consistency of this factor.
- Items like cope14new (0.6241 on factor 2) and cope23new (0.6048 on factor 2) also showed strong, meaningful loadings, indicating that these items were reliable indicators of their designated coping dimension.
- The item cope45new showed a strong loading of 0.6073 on factor 1, further demonstrating that the factor structure was coherent.

Some items exhibited negative loadings; for instance, cope1new displayed a factor loading of –0.4554 on factor 1. Negative loadings are theoretically justifiable when items assess the opposite end of a coping dimension. For example, an item that signifies disengagement may inherently load negatively on a factor that represents active coping. This trend illustrates the multidimensional nature of coping responses, where certain strategies are mutually exclusive.

**Uniqueness values:** Uniqueness denotes the proportion of an item’s variance that remains unexplained by the factor model. Lower uniqueness values are desirable as they suggest that a significant portion of an item’s variance is explained by its corresponding factor. In this research, the majority of items exhibited acceptable uniqueness. For instance:

- cope12new had a uniqueness of 0.5537, meaning nearly half of its variance was explained by the factor it loaded on.
- Items such as cope45new (0.6312) and cope23new (0.6358) also showed desirable levels of explained variance.

A few items exhibited greater uniqueness, including cope42new (0.8412) and cope2new (0.8446). Elevated uniqueness implies that these items were not as effectively represented by the factor model, which may suggest that they address more distinct, culturally specific elements of coping or that their phrasing might require adjustment for this context.

In general, most items showed robust factor loadings and satisfactory uniqueness, reinforcing the construct validity of the Brief COPE within this sample.

**Interpretation of factor structure:** The three-factor solution identified in the CFA affirmed that the theoretical framework of the Brief COPE was predominantly maintained among informal caregivers in Uganda. Although the original instrument comprises a greater number of subscales (15 in total), the aggregation of items into three substantial factors corresponded with existing literature, indicating that coping responses frequently group into problem-focused, emotion-focused, and avoidant categories. The significant loadings observed across these factors illustrated that caregivers consistently distinguished between different coping strategies. For instance, items associated with active coping and planning were grouped together, whereas those assessing denial or disengagement constituted a distinct dimension. This trend provided support that caregivers in Uganda understood coping in ways that align with global theoretical frameworks, albeit potentially influenced by local cultural and socioeconomic contexts.

**Cultural validity:** The findings underscored that the translated and culturally modified version of the Brief COPE maintained its conceptual integrity and framework. Notably, the CFA validated that the coping constructs assessed by the instrument were pertinent and identifiable to Ugandan informal caregivers. This aspect is essential in cross-cultural research, as instruments created in Western settings may not necessarily convey meaningful interpretations to non-Western groups without thorough adaptation and validation.

**Implications for use:** The CFA results presented in Table 4, along with the findings of high internal consistency and test–retest reliability, indicate that the Brief COPE serves as a reliable tool for evaluating coping mechanisms in Uganda. The validated factor structure endorses its application in both research and practical contexts, including the identification of prevalent coping styles among caregivers, the assessment of psychosocial intervention effects, and the development of culturally appropriate mental health programs.

Future research may expand upon these results by performing multi-group CFAs to investigate the stability of the factor structure across different subgroups (for instance, male versus female caregivers or rural versus urban environments) or by analyzing whether specific coping domains are particularly highlighted within various cultural or religious groups.

## Discussion

The results of this research established that the 60-item Brief COPE is a strong, dependable, and valid tool for evaluating coping strategies among informal caregivers of cancer patients in Uganda. The high internal consistency (Cronbach’s α = 0.91–0.92 across key subscales) and robust test–retest reliability (*r* = 0.89) indicated that the instrument offers stable and consistent measurements of coping responses over time. Additionally, the CFA results further confirmed its construct validity by verifying that the factor structure of the tool is coherent and pertinent for this demographic.

This validation significantly contributes to the sparse literature on culturally adapted psychological assessments for caregivers in sub-Saharan Africa. Prior research in Uganda primarily examined the Brief COPE among caregivers of HIV/AIDS or chronic illness patients; this study is one of the first to rigorously evaluate it among informal cancer caregivers—a demographic recognized for experiencing substantial psychological and emotional challenges.

The sociodemographic results underscored crucial contextual elements. In this study, caregivers were mainly women, a considerable number of whom participated in informal or subsistence farming and had low median incomes. Cultural roles influenced the ways in which men and women participated in caregiving and obtained information regarding patient conditions, which had a direct impact on their coping mechanisms. The notable disparities in cancer stage awareness and the caregiver–patient relationship emphasize the necessity of taking gender dynamics and family structures into account when analyzing coping styles.

**Relevance for hospital-based administration:** Within hospital environments, the validated Brief COPE serves as a practical and user-friendly instrument that health professionals, psycho-oncology units, and social workers can utilize to identify coping difficulties among caregivers. Its concise and straightforward format, now validated in various local languages and cultural contexts, allows for its integration into standard caregiver evaluations during patient appointments. Research assistants or nurses are capable of administering the tool in waiting areas or counseling rooms without interfering with clinical operations. The back-translation and pilot testing processes guarantee that caregivers with varying levels of literacy can comprehend and complete it with minimal oversight.

Hospitals can utilize the findings to recognize caregivers who are at risk of maladaptive coping strategies—including those who depend significantly on denial, behavioral disengagement, or substance use—and direct them to appropriate support programs. The subscales of the tool enable healthcare providers to identify which coping areas may require enhancement. For instance, low scores in “active coping” or “planning” could indicate a necessity for skills-based interventions or the development of practical support plans.

**Implications for caregivers:** Validated coping mechanisms, such as the Brief COPE, serve to illuminate the often-overlooked burdens faced by caregivers in a systematic manner. By measuring coping strategies, healthcare facilities and researchers are able to track caregiver well-being in a structured way, rather than depending exclusively on informal observations. This body of evidence can inform the creation of caregiver support programs specifically designed for the Ugandan context—which may include psychoeducation workshops, spiritual counseling, peer support groups, or stress management sessions.

Comprehending the predominant coping strategies of caregivers also empowers the caregivers themselves. When caregivers obtain feedback regarding their coping profiles, they are able to contemplate their strengths and areas that require improvement and actively pursue assistance when necessary. For caregivers encountering cultural obstacles in openly discussing stress or mental health, a systematic, standardized tool offers a secure starting point for dialogues concerning stress and emotional strain.

## Conclusion

**Figure 2:**
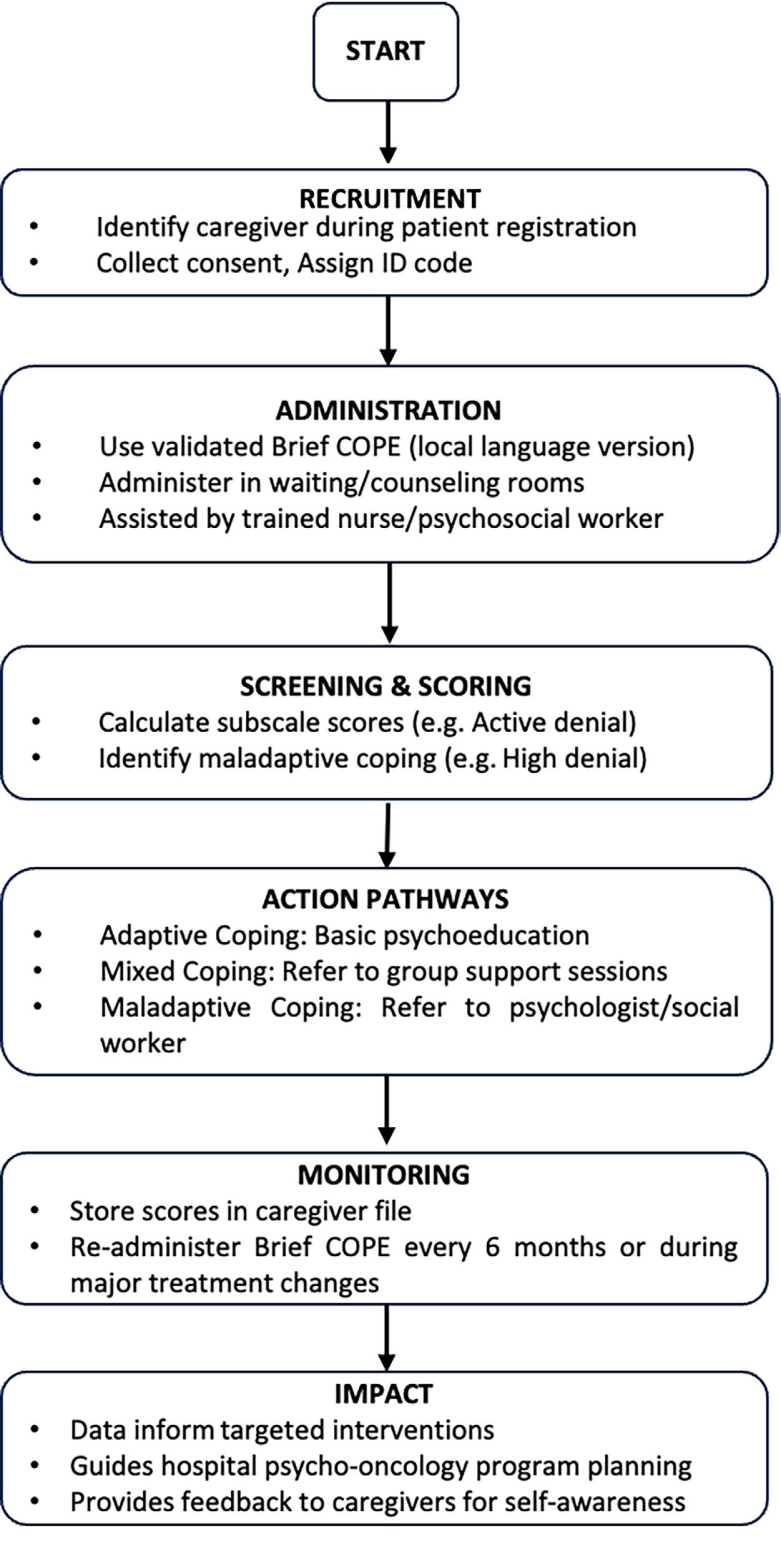
Strategic application of the Brief COPE in hospitals for cancer caregivers.

This research has shown that the 60-item Brief COPE serves as a culturally appropriate and psychometrically robust tool for evaluating coping strategies among informal caregivers of cancer patients in Uganda. Its high internal consistency, outstanding test–retest reliability, and confirmed factor structure indicate that the Brief COPE can effectively capture the responses of caregivers to the complex challenges associated with caregiving in this environment. These results underscore the necessity of adapting and thoroughly validating psychological tools to guarantee their precision and cultural relevance in various contexts. Looking ahead, subsequent studies should broaden the validation of the Brief COPE to include other caregiver demographics throughout Uganda, such as those caring for patients with HIV/AIDS, noncommunicable diseases, and individuals in palliative care. Additionally, longitudinal research is essential to monitor shifts in coping strategies over time and through different stages of caregiving. The Brief COPE can facilitate the creation of focused psychosocial interventions, the development of customized caregiver support initiatives, and the training of healthcare professionals to incorporate caregiver assessments into hospital services (Figure 2). Investigating digital or mobile adaptations of the Brief COPE could further improve its accessibility and reach for caregivers in rural and resource-constrained areas. By rendering caregiver stress visible and quantifiable, the validated Brief COPE establishes a basis for effective support systems that enhance caregiver resilience and ultimately benefit the well-being of both caregivers and the patients they assist.

The flowchart in Figure 2 depicts a systematic framework for incorporating the Brief COPE assessment into standard hospital-based psycho-oncology services aimed at informal caregivers of cancer patients. The illustration demonstrates how hospitals can effectively screen, evaluate, and address the coping needs of caregivers in a structured and culturally appropriate manner.
